# Binding of Estrogenic Compounds to Recombinant Estrogen Receptor-α: Application to Environmental Analysis

**DOI:** 10.1289/ehp.7522

**Published:** 2004-12-09

**Authors:** Arnaud Pillon, Anne-Marie Boussioux, Aurélie Escande, Sélim Aït-Aïssa, Elena Gomez, Hélène Fenet, Marc Ruff, Dino Moras, Françoise Vignon, Marie-Josèphe Duchesne, Claude Casellas, Jean-Claude Nicolas, Patrick Balaguer

**Affiliations:** ^1^INSERM (Institut National de la Santé et de la Recherche Médicale), Unité 540, Montpellier, France; ^2^INERIS (Institut National de l’Environnement Industriels et des Risques), Unité Evaluation des Risques Ecotoxicologiques, Verneuil-en-Halatte, France; ^3^ UMR 5569 “Hydrosciences,” Département des Sciences de l’Environnement et Santé Publique, Faculté de Pharmacie, Montpellier, France; ^4^IGBMC (Institut de Génétique et de Biologie Moléculaire et Cellulaire), Laboratoire de Biologie et Génomique Structurale, Illkirch, France

**Keywords:** aryl hydrocarbon receptor, bioluminescent cell lines, environmental samples, estrogen receptor, xenoestrogens

## Abstract

Estrogenic activity in environmental samples could be mediated through a wide variety of compounds and by various mechanisms. High-affinity compounds for estrogen receptors (ERs), such as natural or synthetic estrogens, as well as low-affinity compounds such as alkylphenols, phthalates, and polychlorinated biphenyls are present in water and sediment samples. Furthermore, compounds such as polycyclic aromatic hydrocarbons, which do not bind ERs, modulate estrogen activity by means of the aryl hydrocarbon receptor (AhR). In order to characterize compounds that mediate estrogenic activity in river water and sediment samples, we developed a tool based on the ER-αligand-binding domain, which permitted us to estimate contaminating estrogenic compound affinities. We designed a simple transactivation assay in which compounds of high affinity were captured by limited amounts of recombinant ER-αand whose capture led to a selective inhibition of transactivation. This approach allowed us to bring to light that water samples contain estrogenic compounds that display a high affinity for ERs but are present at low concentrations. In sediment samples, on the contrary, we showed that estrogenic compounds possess a low affinity and are present at high concentration. Finally, we used immobilized recombinant ER-αto separate ligands for ER and AhR that are present in river sediments. Immobilized ER-α, which does not retain dioxin-like compounds, enabled us to isolate and concentrate ER ligands to facilitate their further analysis.

Endocrine-disrupting compounds (EDCs) are a newly defined category of environmental contaminants that interfere with endocrine system function (Sumpter 1998). Many alterations of the reproductive system observed in the aquatic environment are attributed to the presence of endocrine disruptors. Numbers of studies have focused on compounds that are agonists for the estrogen receptors αand β (ER-αand ER-β) (Kuiper et al. 1998; Paris et al. 2002). These compounds include a wide range of molecules, such as natural or pharmaceutical estrogens, alkylphenols, organochlorine pesticides, and phthalates, that exhibit different binding affinities. Natural and pharmaceutical estrogens have high affinity (dissociation constant *K*_d_ < 1 nM) for ERs, whereas the other groups of molecules display lower affinity (*K*_d_ > 10 nM) and are called xenoestrogens. Furthermore, compounds such as polycyclic aromatic hydrocarbons (PAHs) or dioxin mediate estrogen responses by binding the aryl hydrocarbon receptor (AhR), which in turn forms a complex with ERs (Ohtake et al. 2003). Other compounds, such as hydroxy-PAHs, might bind ERs and AhR (Fertuck et al. 2001).

Sewage treatment plants (STPs) receive a large spectrum of molecules from domestic, agricultural, and/or industrial wastes that are not totally eliminated during treatment processes. At the STP outlets, a complex mixture of molecules, including incompletely eliminated waste water molecules but also metabolites formed during treatment processes, are finally discharged into rivers. In this context, STP discharges are considered a major source of estrogenic water pollution that may play a role in environmental contamination. Several studies reported a correlation between reproductive abnormalities in fish and exposure to STP effluents (Harries et al. 1999; Jobling et al. 2002; Purdom et al. 1994).

Given the difficulty in identifying all of these EDCs, several authors have attempted to detect estrogenic activity and quantify its potency in water samples by targeting their research on specific molecules such as the natural hormones estrone (E_1_), 17β -estradiol (E_2_), and estriol (E_3_); the synthetic estrogen ethynylestradiol (EE_2_); and/or alkylphenols (Aerni et al. 2004; Baronti et al. 2000; Solé et al. 2000). We and others evaluated overall estrogenic activity in water samples (Aerni et al. 2004; Balaguer et al. 2000; Körner et al. 1999). Analytical fractionation systems combined with *in vitro* biological assays were also developed to identify estrogenic compounds present in water. Desbrow et al. (1998) showed, indeed, that compounds with high affinity (E_2_, E_1_, and EE_2_) are responsible for the major part of estrogenic activity in U.K. effluents. A similar observation was made by Snyder et al. (2001) in water samples taken from mid-Lake Michigan and Lake Mead (USA) and by Cargouët et al. (2004) in water samples taken from the river Seine (France). In river sediment samples, conversely, low-affinity compounds such as alkylphenols might contribute to estrogenic activity (Fenet et al. 2003) even if E_2_ and E_1_ are present (Peck et al. 2004).

The objective of this study was to develop tools for characterizing substances that mediate estrogenic activity in complex mixtures, that is, to determine if the estrogenic compounds were ER activators by direct binding (with high or low affinity) or ER activators by another mechanism of action (AhR activation). Estrogenic activity was evaluated with the MELN cell line (Balaguer et al. 1999). Two complementary methodologies were proposed for complex mixture characterization. The first one enabled the capture of compounds of high affinity for ER-αby limited amounts of ER-αligand-binding domain (LBD); this event led to a selective inhibition of luciferase gene expression in MELN cells. The second method allowed ER ligand separation from other compounds by recombinant ER-αimmobilized on agarose columns. Furthermore, estrogen binding columns, which do not retain compounds interacting with AhR only, enabled us to purify and concentrate ER ligands. Estrogen and dioxin-like activities were followed with specific bioluminescent cell lines, MELN and HAhLP cells, respectively. These methodologies were developed with pure compounds and validated with environmental samples, for their applicability on complex mixtures.

## Materials and Methods

### Materials.

Materials for cell culture were obtained from Invitrogen (Cergy-Pontoise, France). Luciferin and isopropylthiogalactopyranoside were purchased from Promega (Charbionnières, France). E_2_, E_1_, E_3_, genistein, coumestrol, α-zearalanol, zearalenone, androstenediol, phenol red dye, dichlorodiphenyl-dichloroethylene (DDE), dioxin, nonylphenol mixture (NPm, ring and chain isomers), 4n-nonylphenol (4-NP), bisphenol A (BPA), and 4-*tert*-octylphenol (OP) were purchased from Sigma Chemical Co. (St. Louis, MO, USA). These effectors were dissolved in dimethyl sulfoxide at 10^−2^ M. [^3^H]-E_2_ (specific activity, 41.3 Ci/mmol) was purchased from NEN Life Sciences Products (Paris, France).

### Plasmids.

Recombinant ER-αwas produced with hER-αLBD(Lys_302_ → Pro_552_) Cys_(381,417,530)_ → Ser triple mutant in fusion with six histidine residues plasmid (as described by Gangloff et al. 2001). Cytochrome P450 1A1–luciferase (CYP1A1-Luc) plasmid was a gift from J.M. Pascussi and P. Maurel (INSERM, U632, Montpellier, France).

### Generation of stably transfected reporter cell lines.

The stably transfected luciferase reporter cell lines (MELN and HAhLP) were obtained as previously described (Balaguer et al. 2001), and the ligand-inducible luciferase expressing clones were identified with a photon-counting camera (NightOWL LB 981; Berthold Technologies, Bad Wildbad, Germany). Briefly, to obtain MELN cells, we transfected ER-α–positive breast cancer MCF-7 cells with the estrogen-responsive gene ERE-βGlob-Luc-SVNeo (Balaguer et al. 1999). Selection of resistant clones by geneticin was performed at 1 mg/mL. The most E_2_-responsive clone was isolated and called MELN 4.1. Basal MELN cell activity was around 15% of maximal activity (10 nM E_2_).

The dioxin reporter cell line was obtained by transfecting HeLa cells with CYP1A1-Luc and pSG5-puro plasmids. Selection of resistant clones by puromycin was performed at 0.5 μg/mL. The most dioxin-responsive clone was denominated HAhLP 1.15. Basal HAhLP cell activity was around 20% of maximal activity (10 nM dioxin).

### Cell culture conditions.

For strain cultures, cells were grown in phenol red containing Dulbecco’s modified Eagle medium, 1 g/L glucose, supplemented with 5% fetal calf serum (FCS), and 1% antibiotic (penicillin/streptomycin) in a 5% CO_2_ humidified atmosphere at 37°C. Because of phenol red and FCS estrogenic activity, *in vitro* experiments were achieved in phenol red–free medium supplemented with 6% dextran-coated charcoal (DCC)–treated FCS (test culture medium).

### Cell luciferase assay.

Cells were seeded at a density of 5 × 10^4^ cells/well in 96-well white opaque tissue culture plates (Becton Dickinson, Le Pont de Claix, France) in 150 μL test culture medium. Compounds to be tested were prepared 4× concentrated in the same medium and 50 μL was added per well 8 hr after seeding. Cells were incubated with compounds for 16 hr. Experiments were performed in quadruplicate and repeated twice. At the end of incubation, effector containing medium was removed and replaced by 3 × 10^−4^ ration, luciferin diffuses into the cell and produces a luminescent signal that is stable from 5 min on. It is approximately 10-fold less intense than a signal after cell lysis would be, but it is perfectly stable for several hours. The 96-well plate was then introduced in a microplate luminometer (Centro LB 960, Berthold Technologies), and intact living cell luminescence measured for 2 sec. Results are expressed as a percentage of maximum luciferase activity. The maximum value, taken as 100, was obtained in the presence of 10 nM E_2_ and dioxin in MELN and HAhLP cell media, respectively. The basal activity (in the absence of ligands) is 15 and 30% of the maximal activity for MELN and HAhLP, respectively. For each estrogenic compound, estrogenic potency corresponding to the concentration yielding half-maximum activity (EC_50_ value) and relative transactivation potency (RTP) were calculated {RTP = [EC_50_ (E_2_)/EC_50_ (test compound)] × 100}. EC_50_ values were evaluated using Graph-Pad Prism statistics software (version 4.0; GraphPad Software Inc., San Diego, CA, USA).

### Environmental samples.

Surface water and sediments were sampled at site U on the Seine watershed (Fenet et al. 2003). Site U is subjected to high inputs of pollutants from an STP in an urban area. Surface water was collected in January 2000 and extracted within 48 hr to minimize bacterial sample degradation. Twenty liters were filtered through a solvent-rinsed Whatman GF/C filter. The filtrate (5 L/column) was concentrated onto preconditioned (20 mL methanol followed by 20 mL water) 5 g C_18_ solid-extraction (SPE) mini-columns. SPE columns were vacuum dried and stored at −20°C. After thawing at room temperature, columns were extracted with 20 mL methanol followed by 20 mL hexane. Methanol eluate volume was reduced to approximately 10 mL, and concentrates were stored at 4°C. Hexane eluates were evaporated to dryness, and residues were taken up with 10 mL methanol. The water sample was therefore 2,000 times concentrated (20 L water giving 10 mL methanol solution).

Sediments (0–5 cm) were collected in November 1999. They were homogenized and sieved through a 2-mm mesh before lyophilization. Lyophilizates (50 g) were extracted twice with dicholoromethane:methanol (2:1) for 20 and 30 min. Extracts were combined and dried by passing through anhydrous sodium sulfate on glass microfibers. The extracts were concentrated (1:100) in double-distilled water to allow extraction of active compound onto the C_18_ cartridge. Compounds bound to the C_18_ phase were eluted with 5 mL methanol. Thus, 1 mL methanol corresponded to 10 g lyophilized sediment.

Sediment and water methanol extracts were applied to MELN and HAhLP cells at 0.3% (vol/vol) maximal concentration in test culture medium, and luciferase transactivation was measured.

### Recombinant receptor production.

The recombinant ER-αcoding plasmid was transformed in BL_21_ DE_3_ electrocompetent *Escherichia coli* cells using the Promega procedure, and the resulting bacteria were plated in ampicillin plates. One colony, after a pre-growth step, was inoculated in 1 L 20% sucrose solution (50% wt/vol) containing Luria Bertani media (10 g/L bactotryptone, 5 g/L bactoyeast extract, 10 g/L NaCl; pH 7.5), until a 0.1 optical density (OD_600nm_) was reached. Cells were then amplified up to OD_600nm_ = 0.2 under 300 rpm agitation and at 37°C. From this point, temperature slowly reached 15°C (in 3 hr), and recombinant receptor synthesis was induced with 0.6 mM isopropylthiogalactopyranoside under the same agitation for 16 hr. Final OD_600nm_ was about 1.4. Cells were centrifuged at 4,000 rpm at 4°C for 40 min. They were homogenized in 100 mL lysis buffer (20 mM Tris HCl, pH 8, 100 mM NaCl, 10% glycerol, 10 mM MgCl_2_, 1 mM MnCl_2_, 1 mg/mL lysozyme, 5 mM β-mercaptoethanol, 1 mg/mL Sigma protease inhibitor cocktail, 14,000 U DNase, and 0.1% Nonidet P40) by rolling at 4°C for 2 hr. Cells were sonicated for 15 min (amplitude, 40; pulse, 2 sec) and centrifugated at 45,000*g* for 60 min. Ligand-binding analysis was performed on supernatant (supplemented with 20% glycerol). The concentration was around 2.5 μM. The soluble recombinant ER-αwas then frozen at −80°C.

In order to purify recombinant ER-α, 2.5 mL of Ni-NTA-agarose phase (Qiagen, Courtaboeuf, France) was washed with washing buffer [WB: 20 mM Tris HCl, pH 7.5, 300 mM NaCl, 20% glycerol, 0.1 mg/mL charcoal-treated bovine serum albumin (BSA), and 10 mM imidazole] and incubated in a column with 100-mL recombinant receptor solution. After rolling for 16 hr, agarose phase was washed with WB, and the receptor was eluted with 7 mL eluting buffer (EB: 20 mM Tris HCl, pH 7.5, 300 mM NaCl, 20% glycerol, 0.1 mg/mL charcoal-treated BSA, and 100 mM imidazole). Protein presence and concentration of each elution fraction were evaluated by SDS-PAGE followed by blue stain reagent coloration. The recombinant ER-α–rich elution fraction was supplemented with 30% glycerol to give a 10 μM purified recombinant ER-α solution and frozen at −20°C.

### Ligand-binding analysis experiments.

For saturation ligand-binding analysis and dissociation constant (*K*_d_) determination, 0.1 pmol recombinant ER-αwas incubated with a range of [3H]-E_2_ (41.3 Ci/mmol specific activity) concentrations in the presence or absence of a 300-fold excess of unlabeled E_2_ in a final volume of 500 μL binding buffer (BB: 20 mM Tris HCl, pH 7.5, 5 mM dithioerythreitol, 2 mg/mL BSA). After shaking at 4°C for 16 hr, bound and free ligands were separated by DCC (2% charcoal, 0.2% dextran in BB). The mixture was left on ice for 2 min and then centrifuged at 3,000 rpm and 4°C for 2 min. Supernatant [^3^H] radioactivity was liquid scintillation counted (LS-6000-SC, Beckman-Coulter, Roissy, France). The *K*_d_ was calculated as the free concentration of radio-ligand at half-maximal specific binding by fitting data to the Hill equation and by linear Scatchard transformation.

For relative binding affinity (RBA) determination, 0.75 pmol recombinant ER-αwas incubated with 2 nM [^3^H]-E_2_ and increasing concentrations of competitors (xenoestrogens or E_2_), in a final volume of 500 μL BB. Experiments were performed as described above in duplicate and repeated twice. For each competitor, the concentration required to inhibit specific E_2_ binding by 50% (IC_50_) was determined as the competitor concentration required to inhibit specific radioligand binding by 50%. IC_50_ values were evaluated using Graph-Pad Prism statistics software. Specific RBA was calculated as the ratio of IC_50_ values of E_2_ to competitor. The RBA value for E_2_ was arbitrarily set at 100.

### Inhibition test of MELN activation.

MELN cells were seeded in 96-well white opaque tissue culture plates as described above. In separate tubes, estrogenic compounds at nonsaturating concentration mediating about 80% MELN cell activity or 0.1% methanol extract for environmental samples, were prepared 4×-concentrated and preincubated with the same volume of 4×-concentrated purified recombinant ER-α(1–100 nM final concentration) in test culture medium at 4°C for 16 hr. Medium from MELN cell culture plates was then removed and replaced by 100 μL of test culture medium supplemented with the same volume of preincubation medium. After 6 hr incubation at 37°C, luciferase activity was determined.

### Purification of estrogenic compounds by immobilized recombinant receptor.

Ten nanomoles of recombinant ER-αwere immobilized on 500 μL Ni-NTA-agarose phase. Estrogenic compounds were prepared in 500 μL WB at different nonsaturating concentrations in regard to their respective estrogenic activities (0.1 nM for E_2_; 3 nM for E_3_, E_1_, and α-zearalanol; 100 nM for Δ5-androstenediol, genistein, OP, and BPA; 300 nM NPm; 10 μM for phenol red) and added to the immobilized receptor. After rolling for 16 hr, flow-through and three 500-μL washings with WB were collected. Liganded receptor was then eluted with 3 × 500 μL EB, and eluate was heated at 65°C for 30 min in order to denature recombinant ER-α. Collected fractions were diluted 10-fold in culture medium before their relative estrogenic activity was evaluated with MELN reporter cells.

Environmental samples (50 μL sediment extract diluted 30-fold in WB; final volume, 1.5 mL) were added to the immobilized receptor. Flow-through (1.5 mL), washing, and elution fractions (500 μL) were collected. Precolumn and flow-through fractions were diluted 10-fold in culture medium before their relative activities were evaluated with MELN and HAhLP reporter cells. Washing and elution fractions were diluted 30-fold in culture medium to take into account the fact that they were 3× concentrated.

## Results

### Recombinant ER-α.

**Figure 1:**
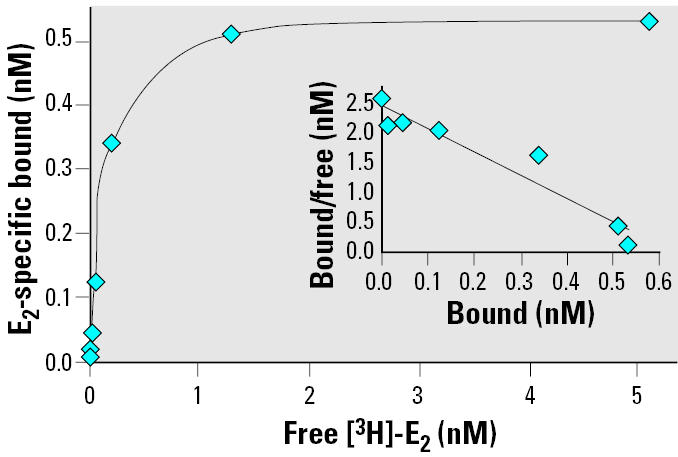
E_2_ binding ability of recombinant ER-α. Binding of [^3^H]-E_2_ to recombinant ER-αwas performed in the presence or absence of non-radioactive E_2_. Unbound radioligand was removed as described in “Materials and Methods,” and specific bound radioligand concentration was calculated after nonspecific bound counts were subtracted from total bound counts. (*Inset*) Linear Scatchard transformation of specific binding giving a *K*_d_ of 0.25 nM for recombinant ER-α.

**Figure 2:**
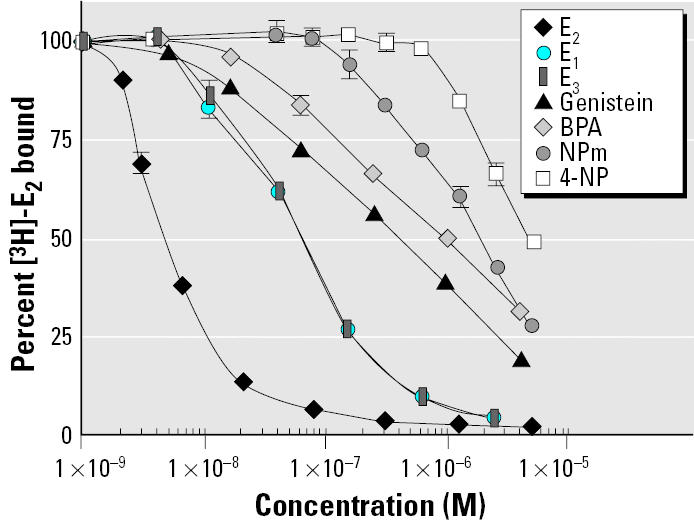
Competitive binding curves of selected nonradioactive chemicals to recombinant ER-α. Recombinant ER-α(0.75 pmol) was incubated with 2 nM [^3^H]-E_2_ together with increasing concentrations of unlabeled test chemicals and incubated in 500 μL at 4°C for 16 hr as described in “Materials and Methods.” The percentage of [^3^H]-E_2_ binding (100% in the absence of unlabeled test chemical) was expressed as a function of the molar concentration of tested compound (mean ± SEM of quadruplicates). IC_50_ values and RBAs are shown in Table 1.

In order to obtain large amounts of ER-α, we decided to produce a mutant ER that would be highly expressed in bacteria. Three of its cysteine residues (381, 417, 530) were mutated into serine residues, which circumvented aggregation and denaturation problems. The mutant protein bound E_2_ with wild-type affinity but had limited transcriptional capacity (Gangloff et al. 2001). Because it had never been tested for its affinity toward xenoestrogens, we tested it by ligand binding ability. *K*_d_ for E_2_ was 0.25 nM, which is close to wild-type ER-αvalue (Figure 1). We also estimated competitor IC_50_ values in our binding conditions by the concentration required to inhibit specific 2 nM [^3^H]-E_2_ binding by 50%. In our conditions, IC_50_ value for E_2_ was about 5.9 nM. Tritiated E_2_ was displaced with an excess of cold compounds such as natural estrogens, (E_1_, E_3_), phytoestrogen (genistein), alkylphenols (4-NP, NPm), and BPA (Figure 2). IC_50_ values and specific relative binding affinities (RBAs) are shown in Table 1. The RBA ranking order was E_2_ > E_3_ = E_1_ > genistein > BPA > NPm > 4-NP. The same compounds were tested by whole cell binding in MELN cells. In these cells, the same ranking order was again observed, confirming that recombinant ER-αtriple mutant exhibited the same binding properties as wild-type ER-α(data not shown).

### Dose–response curves of estrogenic compounds in MELN cells.

**Figure 3:**
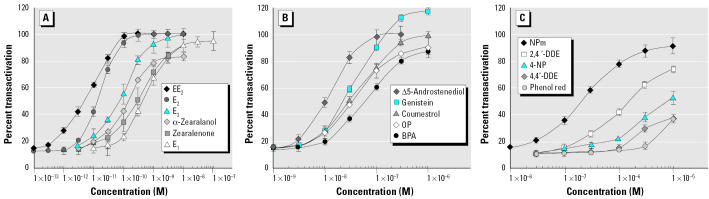
MELN cell luciferase assay of natural estrogens and xenoestrogens. (*A*) Compounds with EC_50_ values from 10 pM to 1 nM. (*B*) Compounds with EC_50_ values from 1 nM to 1 μM. (*C*) Compounds with EC_50_ values > 1 μM. Results are expressed as a percentage of luciferase activity measured per well (mean ± SEM of quadruplicates). The value obtained in the presence of 10 nM E_2_ was taken as 100. EC_50_ values and RTP are shown in Table 2.

A great number of compounds able to activate ER-αwere tested with our MELN cells. We subsequently defined three classes of ligands according to their estrogenic potency (EC_50_ values). The first class was composed of ligands with the highest affinity for ER-α, EC_50_ values ranging from 10 pM to 1 nM. It included EE_2_, a pharmaceutical estrogen, natural estrogens E_2_, E_3_, and E_1_, and mycoestrogen zearalenone and its metabolite α-zearalanol (Figure 3A). The second class was composed of ligands with an EC_50_ values from 1 nM to 1 μM, such as natural estrogen Δ5-androstenediol, phytoestrogens coumestrol and genistein, alkylphenols OP and NPm, and BPA (Figure 3B). Finally, the last class contained compounds such as DDE insecticides, 4-NP, and phenol red dye, which had the lowest affinity for ER-α, with an EC_50_ values of up to 10 μM (Figure 3C). For each tested compounds, the RTP was calculated and reported in Table 2.

**Figure 4:**
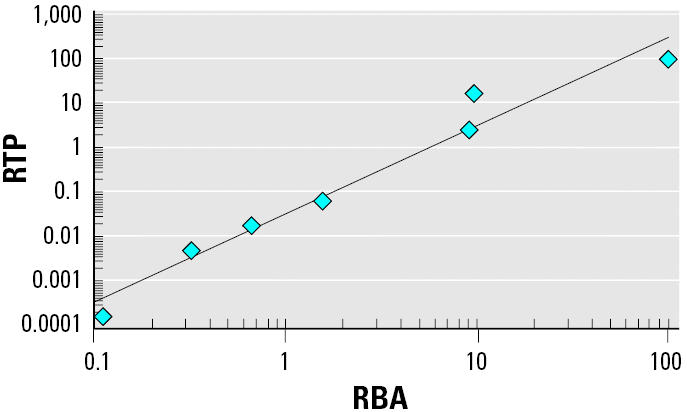
Correlation between binding and transactivation. Linear regression of the RBAs as a function of the RTP of E_2_, E_3_, E_1_, genistein, BPA, NPm, and 4-NP. Values were extracted from Tables 1 and 2. *R*^2^ = 0.9578.

Comparison between binding to recombinant ER-αand transactivation efficiency showed that a good correlation was obtained for all compounds except E_3_ (Figure 4). This linear regression exhibited an *R*^2^ value of 0.9578, but it reached 0.9849 when E_3_ was not taken into account in the correlation analysis (result not shown). E_3_ exhibited an IC_50_ value for recombinant ER-αsimilar to that of E_1_, whereas E_1_ was 7-fold less efficient in MELN cells than was E_3_.

### Inhibition test of MELN activation.

High-affinity estrogens such as E_2_, EE_2_, E_3_, and E_1_ could participate in the estrogenic activity of environmental samples from an urban source. To identify the presence of such compounds having a high affinity for ER-α, present at low concentration and, as a consequence, not easily detectable by classical analytical techniques, we set up a method that we called an inhibition test of MELN activation, in which high-affinity estrogenic compound transactivation of cellular ER-αwas competitively inhibited by limited amounts of recombinant ER-α. Keeping in mind that only free estrogens are able to bind cellular ER-αand activate luciferase expression, we preincubated a group of estrogens with recombinant ER-α. Their binding to recombinant ER-αproduced a diminution of free compound concentration. In a second step, liganded recombinant ER-α preincubation medium was added to cell culture medium and tested for its MELN cell transactivation activity. Sequestered ligands were thus not able to be taken into cells and show estrogen-mediated luciferase activity. The greater the recombinant ER-αconcentration, the more efficient the estrogen capture and inhibition of transactivation.

**Figure 5:**
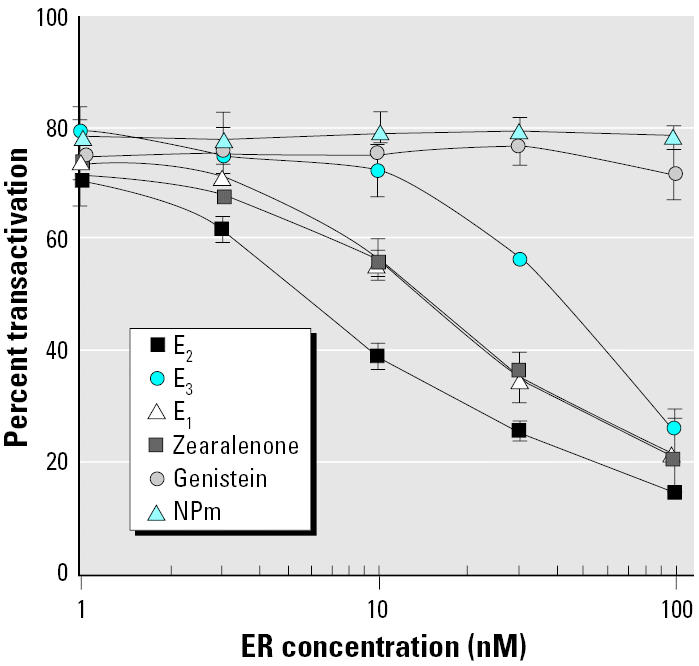
Inhibition test of MELN activation with various estrogens. Luciferase activity induced by E_2_, E_3_, E_1_, zearalenone, genistein, and NPm at concentrations giving about 80% of transactivation was evaluated in MELN cells in the presence of variable amounts of recombinant ER-α(1–100 nM). Results are expressed as the percentage of luciferase activity measured per well (mean ± SEM of quadruplicates). The value obtained in the presence of 10 nM E_2_ was taken as 100.

Capture efficiency of a group of estrogens having very different affinities for ER-α(E_2_, E_3_, E_1_, zearalenone, genistein, and NPm) was determined. As indicated in “Materials and Methods,” they were preincubated with 1–100 nM recombinant ER-αat concentrations necessary to obtain about 80% of MELN cell transactivation. Figure 5 shows that only compounds exhibiting a high affinity for ER-αwere captured and therefore yielded to an apparent inhibition of transactivation activity. E_3_, E_1_, and zearalenone were captured less efficiently than was E_2_, and compounds with lower affinity such as genistein and NPm showed only a slight or no inhibition even at high recombinant ER-α concentration. Although apparent affinities of E_1_ and E_3_ for recombinant ER-αwere similar (Figure 2), less efficient ER-αinhibition of E_3_ MELN transactivation was observed. It could reflect the 7-fold greater efficiency of E_3_ to transactivate cellular ER-α(Figure 3A).

### Purification of estrogens by immobilized recombinant ER-α.

**Figure 6:**
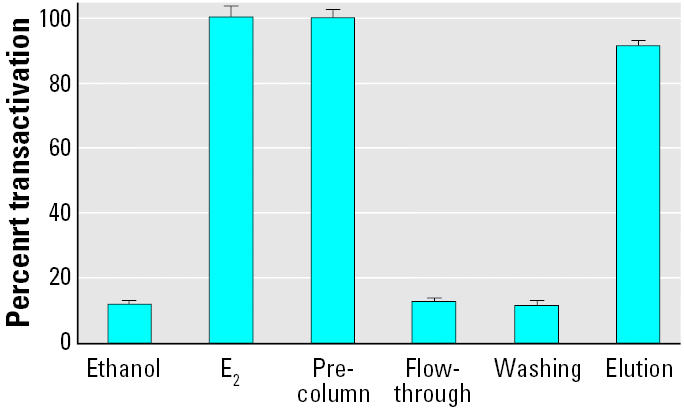
MELN cell luciferase assay of fractions obtained after E_2_ purification on recombinant ER-α agarose column. Recombinant ER-α(10 nmol) was prefixed on 100 μL Ni-NTA-agarose phase. E_2_ (0.1 nM, i.e., 500 fmol) was added, and 500 μL fractions (flow-through, washing, and elution) were collected. The percentage of transactivation of MELN cell luciferase was then measured (values in Table 3). Results are expressed as a percentage of luciferase activity measured per well (mean ± SEM of quadruplicates). The value obtained in the presence of 10 nM E_2_ was taken as 100.

**Figure 7:**
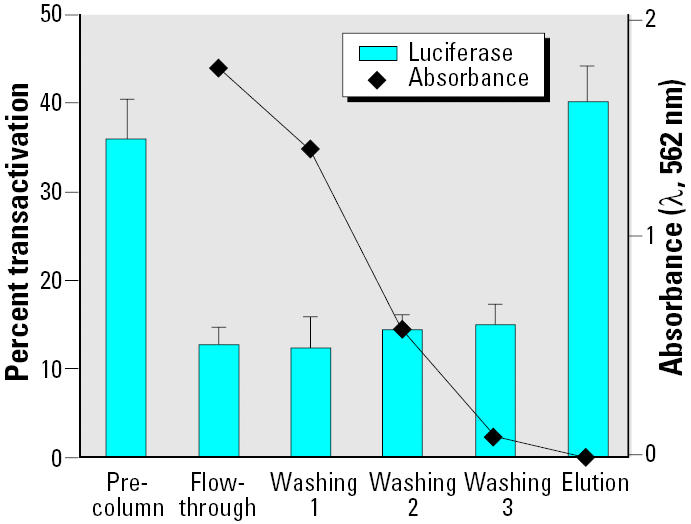
MELN cell luciferase assay of fractions obtained after purification of phenol red on recombinant ER-αagarose column. Recombinant ER-α (10 nmol) was prefixed on 100 μL Ni-NTA-agarose phase. Phenol red (10 μM, i.e., 50 nmol) was added, and 500 μL fractions (flow-through, washing, and elution) were collected. The percentage of trans-activation was then measured (values in Table 3). The left ordinate expresses the percentage of luciferase activity measured per well (mean ± SEM of quadruplicates). The value obtained in the presence of 10 nM E_2_ was taken as 100. The right ordinate expresses phenol red absorbance (λ, 562 nm) of collected fractions.

As described above, low ER-αconcentrations would only bind high-affinity estrogens. On the other hand, recombinant ER-αimmobilized at micromolar concentration was able to capture all estrogenic compounds present in environmental samples. Recombinant ER-αwas immobilized on Ni-NTA-agarose, incubated with different estrogenic compound, and column treated as described in “Materials and Methods.” Figure 6 shows a typical diagram obtained with E_2_ by evaluating estrogenic activity of all the collected fractions with the MELN cell luciferase assay. Table 3 clearly demonstrates the efficiency of capture and the good recovery of various estrogenic compounds. Another application is the purification of estrogenic compounds from a mixture. The estrogenic compound present in phenol red (Bindal et al. 1988) was efficiently separated from the dye by immobilized ER-α(Figure 7).

### Dose–response curves of sediment and water extracts in MELN cells.

**Figure 8:**
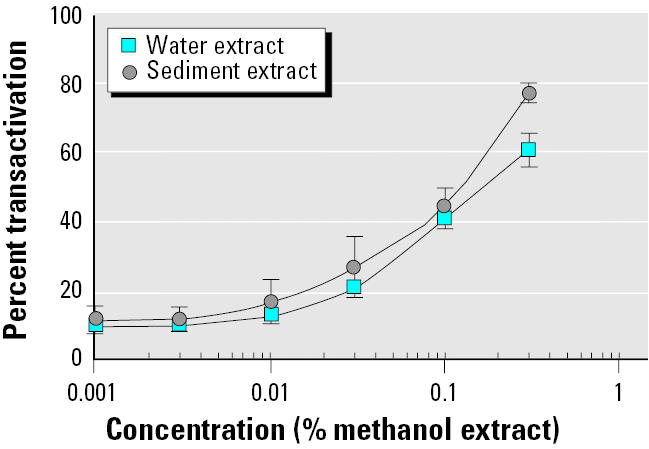
MELN cell luciferase assay of water and sediment extracts. Results are expressed as a percentage of luciferase activity measured per well (mean ± SEM of quadruplicates) as a function of methanol extract percentage. The value obtained in the presence of 10 nM E_2_ was taken as 100.

Estrogenic activities of sediment and water extracts of river Seine in an urban site were evaluated with the help of MELN cell luciferase assay (Figure 8). Sediments and water methanol extracts were applied to MELN cells, and luciferase transactivation was measured. When 0.15% water methanol extract was applied to MELN cell culture medium, a transactivation signal equivalent to that obtained with 15 pM E_2_ was observed (E_2_ EC_50_ value). E_2_ equivalence in water methanol extract was therefore 10 nM. Taking into account the 2,000×-concentrated methanol extract, as described in “Materials and Methods,” estrogen concentration in water was evaluated to 5 pM E_2_ equivalents (E_2_eq).

When 0.12% sediment extract was applied to cell culture medium, a transactivation signal equivalent to that obtained with 15 pM E_2_ was observed. E_2_ equivalence in sediment extract was therefore 12.5 nM. The whole sediment extract (5 mL from 50 g sediment) contained 62.5 pmol E_2_eq, and estrogen concentration in sediment was 1.25 pmol E_2_eq/g. In a previous work (Fenet et al. 2003), we showed that the estrogenic activity in sediments could be explained in great part by the alkylphenol concentration. On the contrary, alkylphenol concentration was too low to contribute to the observed river water estrogenic activity. We therefore hypothesized that other compounds, such as natural and synthetic hormones, could contribute in the overall water activity (Fenet et al. 2003).

### Inhibition test of MELN activation with water and sediment extracts.

**Figure 9:**
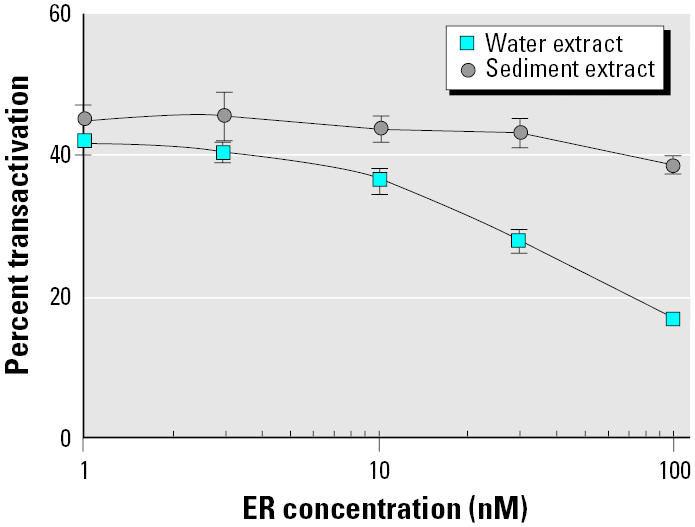
Inhibition test of MELN activation with water and sediment extracts. Induction of luciferase activity by 0.1% water and by sediment methanol extract in culture medium was evaluated in MELN cells in the presence of variable amounts of recombinant ER-α(1–100 nM). Results are expressed as a percentage of luciferase activity measured per well (mean ± SEM of quadruplicates). The value obtained in the presence of 10 nM E_2_ was taken as 100.

In order to confirm the above hypothesis, we performed an inhibition test of MELN cell activation with water and sediment of the same environmental site (Figure 9). Methanol extracts (0.1% vol/vol of test culture medium) corresponding to 10 pM and 12.5 pM E_2_eq for water and sediment, respectively, were added to cells in the presence of variable amounts of recombinant ER-α. Dose–response curves clearly identified the presence of high-affinity compounds in water samples because inhibition of transactivation was great with water extracts, whereas it was small with sediment extracts even at 100 nM recombinant ER-α. Similar results were obtained with a great number of water and sediment river samples (results not shown). Thus, different compositions in high- and low-affinity estrogenic compounds in river water and sediments are evidenced with our assay.

### Purification of sediment extract estrogenic compounds by immobilized recombinant ER-α.

**Figure 10:**
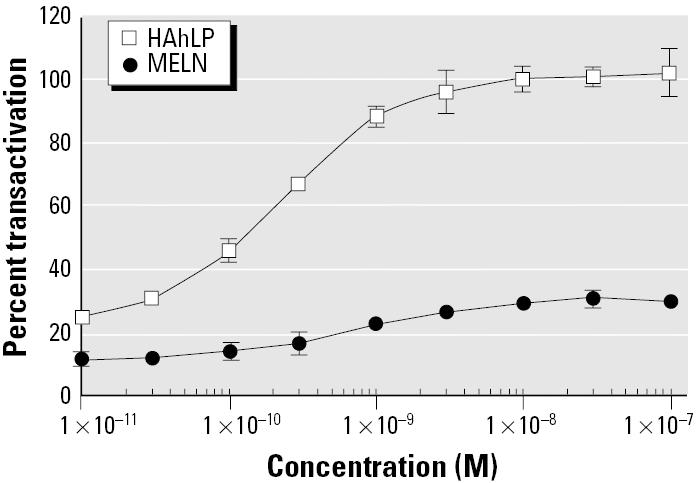
Induction of luciferase activity by dioxin in HAhLP and MELN cell lines. Results are expressed as a percentage of luciferase activity measured per well (mean ± SEM of quadruplicates). The values obtained in the presence of 10 nM dioxin and E_2_ with HAhLP and MELN cells, respectively, were taken as 100. The basal signal was 20 and 15% for HAhLP and MELN cells, respectively.

**Figure 11:**
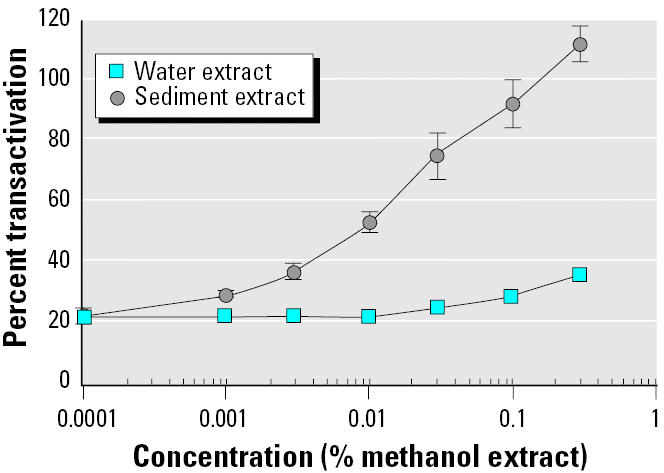
Induction of luciferase activity by water and sediment extracts in HAhLP cell line. Results are expressed as a percentage of luciferase activity measured per well (mean ± SEM of quadruplicates). The value obtained in the presence of 10 nM dioxin with HAhLP cells was taken as 100%.

In river sediments, various substances were shown to bind to AhR (Michallet-Ferrier et al. 2004). These compounds can be dioxins, some PAHs, polychlorinated biphenyls (PCBs), and various pesticides. In order to characterize AhR activity of the sediment sample, we established AhR-responsive HeLa cell lines (HAhLP). In HAhLP cells, dioxin induced luciferase expression with an EC_50_ value of 0.2 nM (Figure 10). As we have already shown (Balaguer et al. 1999), dioxin was also able to partially activate MELN cells (Figure 9). This estrogenic activation by AhR ligands is mediated by a ternary complex (ER-α, AhR, and Arnt) in MCF-7 cells (Ohtake et al. 2003). As expected, sediment extract had a strong dioxin-like activity (0.2 nmol dioxin equivalents/g sediment), whereas water extract had a weak AhR activity (Figure 11). This strong dioxine-like activity could be due to PAHs widely found in river sediments (Hilscherova et al. 2001; Michallet-Ferrier et al. 2004).

**Figure 12:**
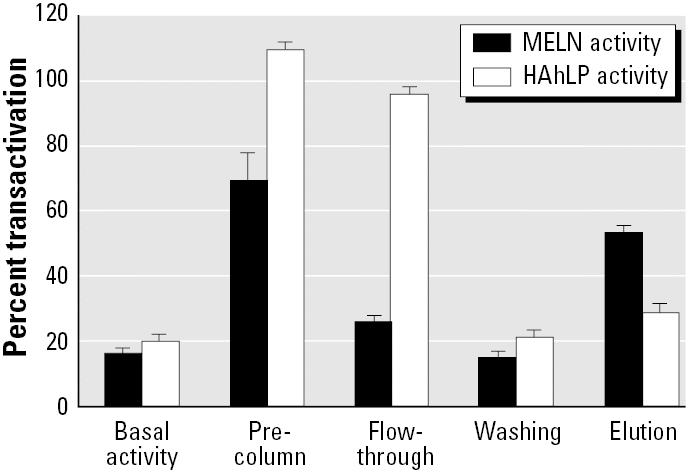
Induction of luciferase activity in MELN and HAhLP cells by column fraction obtained after purification of sediment extract on recombinant ER-αagarose column. Ten nanomoles of recombinant ER-αwere prefixed on 100 μL Ni-NTA-agarose phase. Sediment extract (50 μL methanol extract 30-fold diluted in WB) was added. Flow-through (1.5 mL), washing, and elution (500 μL) fractions were collected and the percentage of transactivation measured. Dilution factors used with both cell lines were 10 for precolumn and flow-through and 30 for washing and elution fractions. Results are expressed as a percentage of luciferase activity measured per well (mean ± SEM of quadruplicates). The values obtained in the presence of 10 nM dioxin and E_2_ with HAhLP and MELN cells, respectively, were taken as 100.

The biological activities of sediment extracts could be due to compounds able to bind each receptor (ER-αand AhR) or a mixture of compounds able to bind only one of the two receptors. In order to address this problem, we used the recombinant ER-αcolumn to separate ER ligands from other compounds. Sediment extracts were applied to the column and ER-α, and AhR activities were measured in the different fractions (Figure 12). Most of the estrogenic activity was in the elution fraction. A small part was not retained by the column and was in the flow-through fraction. This weak estrogenic activity may be due to AhR ligands because most AhR activity was in the flow-through and wash fractions. A small part of AhR activity was in the fraction eluting with recombinant ER-α. Because estrogens did not activate luciferase expression in the AhR-responsive cell line (results not shown), we conclude that some compounds have a double activity (estrogenic and dioxin-like).

## Discussion

Environmental estrogenic activity is mediated by a wide variety of compounds that may be differentially distributed in water or sediments. These chemicals include a wide range of molecules from natural, pharmaceutical, agricultural, or industrial origin. Nevertheless, STP effluents are considered a major source of estrogenic water pollution that may play a role in environmental contamination. High-affinity compounds for ERs, such as natural or synthetic estrogens, as well as low-affinity compounds, such as alkylphenols, phthalates, and hydroxylated PCBs, have been identified in river water and sediments samples. Given the difficulty in identifying all of these EDCs, numerous authors attempted to detect and quantify the estrogenic potency of water samples by targeting their research on specific molecules (Aerni et al. 2004; Baronti et al. 2000; Solé et al. 2000). Analytical extraction systems combined with *in vitro* biological assays were also developed to identify estrogenic compounds present in water and sediments (Cargouët et al. 2004; Desbrow et al. 1998; Fenet et al. 2003; Peck et al. 2004; Snyder et al. 2001).

Nevertheless, it is difficult to assert if the observed effects are due to compounds of low or high affinity before they are identified. We developed a tool using a recombinant ER-α LBD in order to trap estrogenic molecules. We first used it to characterize the affinity of unknown estrogenic compounds. It is a simple assay in which compounds of high affinity were captured by limited amounts of recombinant ER-αleading to a selective inhibition of transactivation in estrogen-responsive cells. This approach allowed us to discriminate between compounds present at low concentration but displaying a high affinity for ER, and compounds present at higher than 10 nM concentration but with a corresponding lower affinity. Furthermore, this recombinant ER-α, immobilized on columns, can be used to extract and concentrate all xenoestrogens, independently from their affinity. In a complex mixture, this procedure would facilitate their analytical identification.

A second best-characterized pathway for endocrine disruption is ligand binding to AhR. This would lead to endocrine-disrupting effects by activating AhR-responsive genes, such as *CYP1A1*, which encodes for cytochrome P450 1A1 involved in endogenous steroid hormone metabolism (Denison and Nagy 2003, Hahn et al. 1996) and/or by modulating ER-responsive gene expression (Ohtake et al. 2003; Wormke et al. 2003). In the aquatic environment, various organic substances were shown to bind AhR. For example, PAHs are known to bind AhR (Billiard et al. 2002), whereas some PAHs and metabolites bind ERs (Charles et al. 2000; Clemons et al. 1998; Fertuck et al. 2001).

Many environmental organic chemicals are hydrophobic and are associated with particulate matter in aquatic ecosystems. Sediments act as both sink and source for these contaminants. They can sorb these chemicals and release them back directly to the food web by ingestion (i.e., benthic organisms) or via resuspension and possible release to the water phase, according to physical-chemical factors and partitioning equilibrium (Gewurtz et al. 2000). In our study, sediments showed both AhR and ER agonistic activities. These activities may be due to *a*) a compound able to bind both ERs and AhR, such as PAHs (Fertuck et al. 2001; Fielden et al. 2000), polybrominated diphenyl ether (PBDE; de Wit 2002), or PCBs (Yoon et al. 2001), or *b*) a mixture of compounds able to bind either one of the two receptors. In order to choose between the two possibilities, we immobilized our recombinant ER-αupon columns to isolate ER ligands from the other compounds and measured ER and AhR activities. It clearly indicated that most AhR ligands did not bind ERs. Altogether, our results indicate that river sediment estrogenic activity is mediated by *a*) low-affinity estrogens that bind only ERs, *b*) most AhR ligands that activate ER-mediated expression through AhR, and *c*) a minority of AhR ligands that activate both ERs and AhR. These hypotheses also address recent findings suggesting the participation of AhR ligands with low estrogenic capacity such as PAHs (Hilscherova et al. 2001; Michallet-Ferrier et al. 2004) and/or PBDEs (Meerts et al. 2001) in estrogenic activities measured in river sediments.

In environmental samples, compounds with high affinity for ER are present mainly in water, whereas medium- or low-affinity compounds are more likely present in sediments. Xenoestrogen trapping mediated by nuclear receptor and coupled to gene expression measurement is more than a screening method. This battery of *in vitro* tests is a powerful, simple, and rapid tool that enables the characterization of compounds present in environmental compartments.

## Figures and Tables

**Table 1:** Binding affinity of various compounds for recombinant ER-α.

Compound	IC_50_ ± SE	RBA
E_2_	5.90 nM ± 1.19	100
E_3_	62.7 nM ± 10.9	9.41
E_1_	65.3 nM ± 10.9	9.04
Genistein	381 nM ± 130	1.55
BPA	889 nM ± 127	0.66
NPm	1.86 μM ± 0.11	0.32
4-NP	5.60 μM ± 1.12	0.11

IC_50_ values were determined from competitive binding experiments performed as described in “Materials and Methods” (Figure 2). Competitor RBAs were calculated as the ratio of IC_50_ values of E_2_ to competitor. RBA value for E_2_ was arbitrarily set at 100.

**Table 2:** Estrogenic potency of tested compounds.

Compound	EC_50_ ± SE	RTP
EE_2_	7.14 pM ± 1.09	246
E_2_	17.6 pM ± 4.61	100
E_3_	100 pM ± 10.8	17.6
α -Zearalanol	135 pM ± 31.2	13.0
Zearalenone	313 pM ± 39.2	5.62
E_1_	694 pM ± 131	2.54
Δ5-androstenediol	13.1 nM ± 5.40	0.13
Genistein	26.7 nM ± 14.7	0.064
Coumestrol	43.5 nM ± 7.91	0.040
4-OP	54.2 nM ± 13.8	0.032
BPA	96.3 nM ± 27.3	0.018
NPm	339 nM ± 126	0.0051
2,4′-DDE	2.74 μM ± 0.92	0.00063
4-NP	11.0 μM ± 2.83	0.00016
4,4′-DDE	25.6 μM ± 11.4	0.00007
Phenol red	36.9 μM ± 23.0	0.00005

EC_50_ values were concentrations required to produce half-maximal induction in MELN cell line, determined from Figure 3. The RTP of each compound was calculated as the ratio of EC_50_ values of E_2_ to compound. RTP value for E_2_ was arbitrarily set at 100.

**Table 3:** Induction of luciferase activity (%) by different fractions of the recombinant ER-αcolumn incubated with natural estrogens and xenoestrogens.

Compound	Precolumn	Flow-through	Washing	Elution
E_2_ (0.1 nM)	95 ± 5	19 ± 4	16 ± 2	91 ± 4
E_3_ (3 nM)	62 ± 3	16 ± 0.5	15 ± 1	60 ± 3
α -Zearalanol (3 nM)	88 ± 2	17 ± 2	16 ± 1	82 ± 6
E_1_ (3 nM)	48 ± 2	19 ± 3	21 ± 3	39 ± 1
Δ5-Androstenediol (100 nM)	93 ± 3	15 ± 0.5	16 ± 0.5	90 ± 3
Genistein (100 nM)	97 ± 5	16 ± 2	17 ± 2	79 ± 2
Coumestrol (100 nM)	71 ± 5	15 ± 3	15 ± 3	66 ± 6
OP (100 nM)	73 ± 3	16 ± 3	15 ± 2	69 ± 2
BPA (100 nM)	70 ± 6	17 ± 2	16 ± 2	70 ± 2
NPm (300 nM)	57 ± 4	17 ± 1	16 ± 2	56 ± 2
Phenol red (10 μM)	36 ± 5	16 ± 2	15 ± 3	40 ± 4

Estrogenic compounds were added to the recombinant ER-α column. The volume of each collected fraction was 500 μL, and their relative estrogenic activity was evaluated with MELN reporter cells. Results are expressed as a percentage of luciferase activity measured per well (mean ± SEM of quadruplicates). The value obtained in the presence of 10 nM E_2_ was taken as 100.
